# Improving Models of Care for Diabetes in Pregnancy: Experience of Current Practice in Far North Queensland, Australia

**DOI:** 10.3389/fpubh.2019.00192

**Published:** 2019-07-19

**Authors:** Anna McLean, Renae Kirkham, Sandra Campbell, Cherie Whitbread, Jennifer Barrett, Christine Connors, Jacqueline Boyle, Alex Brown, Jacqueline Mein, Mark Wenitong, H. David McIntyre, Federica Barzi, Jeremy Oats, Ashim Sinha, Louise Maple-Brown

**Affiliations:** ^1^Cairns Hospital, North Cairns, QLD, Australia; ^2^Wellbeing and Preventable Chronic Disease Division, Menzies School of Health Research, University Drive North, Casuarina, NT, Australia; ^3^Department of Health, Central Queensland University, Cairns, QLD, Australia; ^4^Royal Darwin Hospital, Tiwi, NT, Australia; ^5^Apunipima Cape York Health Council, Bungalow, QLD, Australia; ^6^Top End Health Service, Northern Territory Department of Health, Darwin City, NT, Australia; ^7^Monash Centre for Health Research and Implementation, School of Public Health and Preventive Medicine, Monash University, Clayton, VIC, Australia; ^8^Population Health Research, University of South Australia, Adelaide, SA, Australia; ^9^South Australian Health and Medical Research Institute, Adelaide, SA, Australia; ^10^Wuchopperen Health Service, Cairns, QLD, Australia; ^11^Mater Medical Research Institute, University of Queensland, South Brisbane, QLD, Australia; ^12^Melbourne School of Population and Global Health, University of Melbourne, Carlton, VIC, Australia

**Keywords:** gestational diabetes–mellitus, diabetes in pregnancy, model of care, screening practices, diabetes management, care coordination, access to health care

## Abstract

**Aims:** To map health practitioners' experiences and describe knowledge regarding screening and management of Diabetes in Pregnancy (DIP) in Far North Queensland, Australia.

**Methods:** Mixed methods including a cross-sectional survey (101 respondents) and 8 focus groups with 61 health practitioners. All participants provided clinical care for women with DIP.

**Results:** A wide range of healthcare professionals participated; 96% worked with Indigenous women, and 63% were from regional or remote work settings. Universal screening for gestational diabetes at 24–28 weeks gestation was reported as routine with 87% using a 75 g Oral Glucose Tolerance Test. Early screening for DIP was reported by 61% although there was large variation in screening methods and who should be screened <24 weeks. Health practitioners were confident providing lifestyle advice (88%), dietary, and blood glucose monitoring education (67%, 81%) but only 50% were confident giving insulin education. Electronic medical records were used by 80% but 55% also used paper records. Dissatisfaction with information from hospitals was reported by 40%. In the focus groups improving communication and information technology systems were identified as key areas. Other barriers described were difficulties in care coordination and access for remote women.

**Conclusions:** Communication, information technology systems, coordination of care, and education for health professionals are key areas that will be addressed by a complex health systems intervention being undertaken by the DIP Partnership in North Queensland.

## Introduction

Effective management of diabetes in pregnancy (DIP) is increasingly a public health concern, as rates of this condition continue to rise in Australia and globally (1, 2). DIP includes both diabetes diagnosed during pregnancy termed Gestational Diabetes Mellitus (GDM) and pre-existing Type 1 Diabetes Mellitus (T1DM) and Type 2 Diabetes Mellitus (T2DM). The introduction of the most recent World Health Organization (WHO) and Australasian Diabetes in Pregnancy Society (ADIPS) diagnostic criteria have contributed to an increase in the screening and diagnosis of GDM (3) as has the “epidemic” of T2DM in the general population. Early screening of women considered at high-risk has led to an increase in early diagnosis of GDM, more frequent antenatal appointments and busier clinics.

Australian population groups such as Aboriginal and Torres Strait Islander women and other high risk ethnic groups have higher rates of GDM and pre-existing T2DM compared to Caucasian women (4, 5). In Australia during the 2-year period from 2014 to 2015, 10% of all births recorded in the National Perinatal Data Collection were complicated by DIP. Of these 9% had GDM, and 1% had pre-existing diabetes (6, 7). In the Northern Territory, the rate of GDM in Aboriginal women is close to 16% and the rate of T2DM is 4% (8). Additionally, Indigenous babies are more likely to have pregnancy-related complications and increased care requirements, regardless of the mother's diabetes status (pre-existing diabetes, GDM, or no diabetes) (6).

These women require intensive multidisciplinary care during pregnancy, as well as pre- and post-partum. Well-recognized acute and chronic complications of DIP can be improved with individualized treatment (9), and by improving systems through measures such as implementation of screening practices (10) and standardized models of care (11, 12).

High quality care for women with DIP and their infants requires a range of services provided by multiple specialties including midwives, diabetes educators, dietitians, obstetricians, general practitioners, endocrinologists, pediatricians, and other health workers. Coordination of the care can be challenging, particularly for women in rural and remote regions, and the increasing number of women diagnosed with DIP threatens to stretch health resources (13).

In the Northern Territory (NT) of Australia, a National Health and Medical Research Council funded Diabetes in Pregnancy Partnership commenced in 2012. The project was designed to address the complex issue of optimizing management of women with DIP and to reduce gaps between evidence and practice (14). The NT Partnership is a collaboration between clinicians, researchers, health care services and policy makers. It has established strong relationships with communities and health services, formed an active Clinical Reference Group, developed enhanced models of care for DIP and successfully established the NT DIP Clinical Register in Darwin and Alice Springs regions (15, 16). Initial results indicate a significant increase in reporting of gestational diabetes and an increased awareness and understanding of the disease burden of DIP (15, 16). In 2016 the partnership was expanded to include Far North Queensland (FNQ) with the aim of establishing a DIP Clinical Register and development of enhanced models of care to augment health professionals' capacity for managing DIP (Diabetes In Pregnancy Partnership In North Queensland “DIPPINQ”).

FNQ has a population of approximately 240,000 in the greater Cairns area and 20,000 in Cape York and the Torres Strait Islands. A large proportion of the region's population identify as Aboriginal or Torres Strait Islander: 10% of the Cairns population, 69% of the Torres Strait Islands' population and 52% of Cape York's total population (17). Cairns Hospital is the major referral center for the Cairns and Hinterland Health Service and the Torres and Cape York Hospital and Health Service which in total span ~273 000 square kilometers, slightly larger than the United Kingdom (18). The rate of GDM in the region is 12–14%, much higher than the national average of 5–10% (19, 20). There are 2,500 deliveries per year at the Cairns Hospital. In 2016, when using the WHO criteria for diagnosis of GDM, 14.5% were complicated by hyperglycaemia: 12% of women had GDM, 2% of women had T2DM and 0.5% T1DM (local hospital data). Multiple service providers including Aboriginal Community Controlled Medical Services, the Royal Flying Doctor Service, private General Practice and Queensland Health primary to tertiary care are involved in care provision. Multiple, separate information systems are used by the various providers. The WHO and ADIPS guidelines for DIP have provided standards for the management of DIP in the region since 2015, and a comprehensive Queensland Health Clinical Guideline for GDM updated in 2015 is widely available. However, adherence to guidelines and provision of seamless care for a high-risk population is an ongoing challenge.

Here we describe findings from formative work of the DIPPINQ. The aims of our study were to 1. Describe knowledge among health practitioners regarding screening and management of women with DIP and 2. Map practitioners' experiences providing care for women with DIP to inform future interventions to improve models of care in Far North Queensland.

## Methods

This study used a mixed methods approach to map the knowledge and experiences of health practitioners who provide healthcare for women with DIP and their families in FNQ. Quantitative data from a survey of health practitioners were triangulated with qualitative data from focus groups conducted with health practitioners.

### Health Practitioner Survey

A cross-sectional survey comprising 48 questions (see Supplement 1) was adapted to the FNQ context from earlier work conducted by the Partnership in the NT. The NT survey was informed by a series of regional workshops with stakeholders. Workshop participants identified issues associated with models of care for DIP. The main constructs underpinning the survey included: communication; information technology; care-coordination; logistics and access; knowledge, education, and guidelines (13).

In FNQ it was distributed electronically via a web based site (Survey Monkey) and hard copies were distributed at meetings and workshops between November 2016 and April 2017. One hundred and one health professionals involved in DIP care from all disciplines participated.

Participants were purposively recruited by advocates of the Partnership from a number of partner organizations (government and non-government, Aboriginal Controlled Community Health Organizations, primary to tertiary care providers). Snowball sampling was employed, whereby participants were asked to forward the survey link within their relevant networks.

### Health Practitioner Focus Groups

A phenomenological methodology guided the qualitative aspect of this study. Participants were purposively recruited to participate in focus groups through Partnership networks (as above). No exclusion criteria were placed on eligibility. A total of eight focus groups with 61 health professionals took place between March and May 2017. Five face-to-face groups were held in conjunction with Partnership workshops in Cairns. The remaining three were arranged in outlying regions via teleconference. Each focus group comprised health professionals from the same organization or region (min = 3, max = 11). Participants worked in primary to tertiary health care settings in urban, regional and remote locations of FNQ. The average duration of focus groups was 58 min.

The focus groups were facilitated by members of the research team with expertise in community-based research (JB, SC, CW, RK). Participants provided informed consent and permission for audio-recording and transcription of discussions. Only one participant declined the invitation to participate.

Discussion in the groups was guided by one of two scenarios about a pregnant woman's journey with DIP. These were developed by the research team and considered relevant to different health care settings. Facilitators used an interview schedule to enquire about the method of coordinating appointments and travel, which providers the woman would see, what guidelines were used, how information was communicated and who was responsible for various aspects of the woman's care.

### Data Analysis

Survey data were exported from Survey Monkey and analyzed using Stata version 14 (Stata Corporation, College Station, Texas). Basic frequencies and percentages were reported and comparisons were made between groups in selected answers using Chi-square tests. Open ended responses were thematically coded.

Focus group data were inductively analyzed in NVivo (QSR International Version 10, 2012) by three members of the research team (SC, JB, and RK). Coding structures were cross-checked for accuracy and interpretation of meaning. A second round of coding was undertaken by RK to ensure saturation was reached on main themes and that results reported on provide further insight into survey findings.

### Ethics

Ethics approval for this study was obtained from the Far North Queensland Human Research Ethics Committee (HREC/16/QCH/15).

## Results

### Health Professional Survey

**Figure 1:**
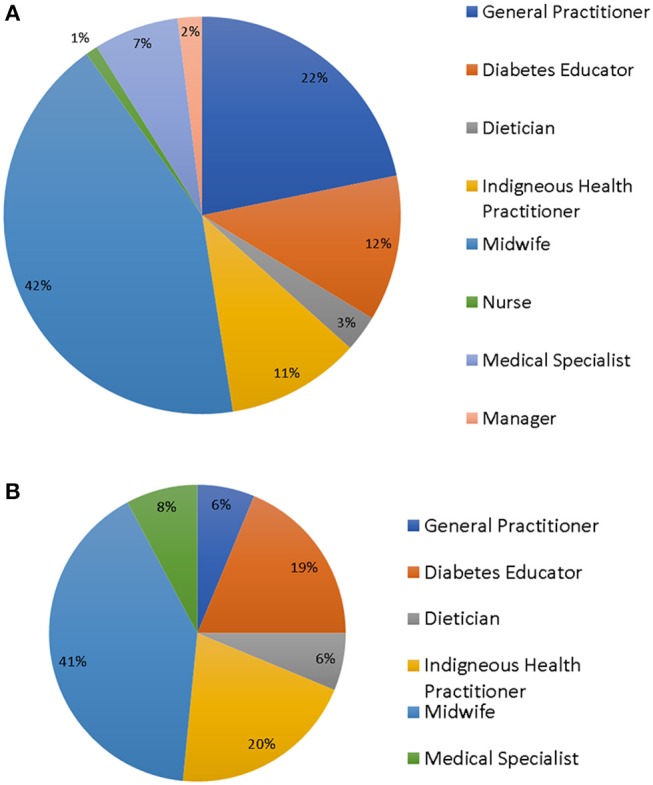
**(A)** Survey participants. **(B)** Focus group participants.

There were 101 survey respondents (see Figure 1), including a range of health professionals (HP) from an even mix of urban, remote, and regional work settings, with midwives being the largest professional group. Ninety-six percent of participants worked with Indigenous women. Of these, 55% had been in their current position for 0–5 years, 18% for 5–10 years, and 28% more than 10 years. Practitioners were not routinely involved in pre-pregnancy counseling, but many were involved in patient care for some time post-partum (Table 1).

**Table 1:** Health Professional Survey: respondent demographics.

**Characteristic**	**Frequency (%)**
**MAIN WORK SETTING**	
Remote	29 (29)
Regional	34 (34)
Urban	24 (24)
Other^*^	12 (12)
**CLIENT BASE**	
Aboriginal women	21 (21)
Torres Strait Islander women	7 (7)
Non-indigenous women	4 (4)
All of the above	68 (68)
**TIME IN CURRENT POSITION**	
<1 year	10 (10)
1–5 years	44 (44)
5–10 years	18 (18)
>10 years	28 (28)
**WHAT PERCENTAGE OF WOMEN WOULD YOU HAVE ALSO SEEN FOR PRE-PREGNANCY COUNSELING?**	
0–20%	67 (83)
20–40%	11 (14)
>40%	3 (3)
**WHAT PERCENTAGE OF WOMEN WOULD YOU ALSO SEE POST-PARTUM?**	
0–20%	34 (42)
2–40%	9 (11)
>40%	38 (47)

* *Total respondents varied per questions and was as follows: Main work setting, n = 99; Client base, n = 100; Percentages of women seen pre-pregnancy and post-partum, n = 81*.

#### Current Screening Practice

**Figure 2:**
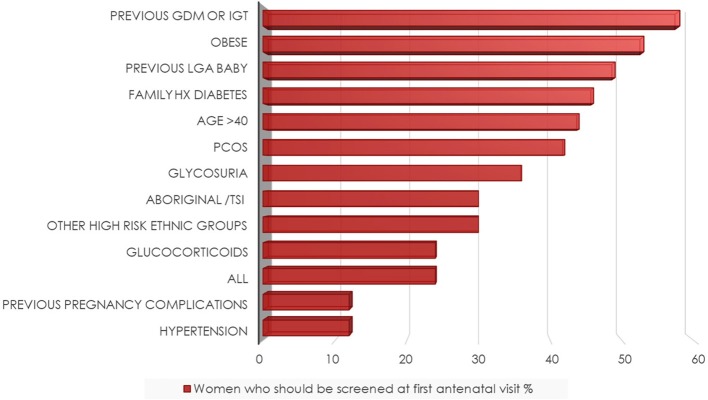
Risk factors^*^ determining screening at the first antenatal visit. ^*^Respondents could choose multiple answers, see question 10 in Supplement 1.

The majority (85%) of respondents reported universally screening for DIP at 24–28 weeks gestation and 87% reported using a 75 g 2 h Oral Glucose Tolerance Test (OGTT) at this time. Routine screening for diabetes in early pregnancy (<24 weeks gestation) was reported by 61% of HP and there was variation in which screening test was used. Fifty two percent reported using a 75 g OGTT, 19% used a HbA1C and 19% a random Blood Glucose Level (BGL) in early pregnancy. Five percent were unsure and 5% did not answer. There was similar discordance in agreement regarding which risk factors would indicate screening was required in early pregnancy, the most common reasons being a previous history of GDM or glucose intolerance, obesity, and previous large baby. Other risk factors including ethnicity were deemed less important (Figure 2).

### Current Screening Practice

**Figure 3:**
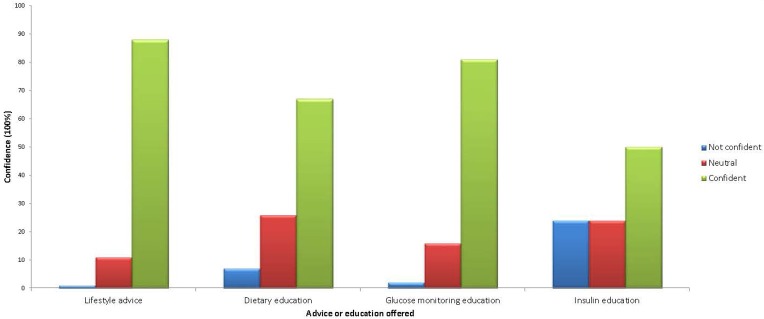
Health professionals' confidence in providing advice or education.

Fifty-eight percent of HP were confident in managing women with DIP (6% reported not being confident and 35% neutral). Results revealed that most were confident providing lifestyle advice, dietary education, and blood glucose monitoring education (88, 67, 81% respectively, were “confident” or “very confident” in these areas) but only 50% were confident in providing information regarding administration and storage of insulin (Figure 3). There was no difference in confidence according to time in job or location of practice (regional vs. remote). However, midwives, dietitians, and diabetes educators reported significantly greater confidence when compared to general practitioners and nurses (74 vs. 43%, *p* = 0.002).

HP reported that they used a variety of resources for their own and patient education, with 63% reporting regular reference to Queensland Health Clinical Guidelines. Their preferences for ongoing education included talks from specialists (32%), online learning modules (30%), and as part of a conference or symposium (22%). Seventy- seven percent of respondents suggested women with DIP would benefit from more education for local health workers.

#### Referrals, Communication, and Care Coordination

Referral methods described were most commonly facsimile (50%), followed by email (34%), phone calls (27%), directly via electronic record (12%), and mail (10%). Most (91%) did not report making referrals to medical specialists, yet 93% did make referrals to allied health specialists. Satisfaction with the referral process and communication from medical and allied health specialists was mostly positive (Table 2). However, only 1% reported being satisfied with written information received from client hospital admissions and the timeliness of information. Respondents reported that medical specialists should be involved in managing women with DIP about the same (56%), more (26%), and much more (11%).

**Table 2:** Health professionals' satisfaction with care coordination.

	**Dissatisfied or very dissatisfied**	**Neutral**	**Satisfied or very satisfied**	**Not applicable**
**SATISFACTION WITH REFERRALS AND COMMUNICATION**				
The process of referring to medical specialists	9 (11)	20 (24)	50 (61)	3 (4)
The communication received back from medical specialists	27 (33)	17 (21)	33 (40)	5 (6)
The process of referring to allied health specialists	7 (9)	20 (24)	54 (65)	2 (2)
The communication received back from allied health specialists	12 (14)	17 (20)	51 (61)	3 (4)
**SATISFACTION WITH SPECIALIST OR HOSPITAL APPOINTMENTS AND ADMISSIONS**				
Written information received from client hospital admissions	29 (37)	50 (63)	1 (1)	0
Timeliness of information received from client hospital appointments or admissions	32 (40)	47 (59)	1 (1)	0
Process of arranging appointments in the nearest hospital or specialist clinic	14 (17)	64 (79)	3 (4)	0

*Total respondents varied per question and was as follows: Satisfaction with referrals and communication to: medical specialists (n = 82) and allied health specialists (n = 83); Satisfaction with specialist or hospital appointments and admissions: Written information (n = 80), Timeliness (n = 80), Process of arranging appointments (n = 81)*.

Telephone or video case conferencing was reported as useful for client care by 83% of those who had used it, however, only 42% reported actually using telehealth. That telehealth should be used more often was reported by 21%, and about the same by (54%). Most used an electronic medical record system (80%) but 55% also still used hand held paper records. Use of the nation-wide “My eHealth” record was not widespread (13%).

Seventy-nine percent thought a DIP Clinical Register in FNQ would be useful. The main benefits were thought to be improved care coordination (62%) with the ability to review care delivered by other providers (53%), offering follow-up screening recall lists (62%), and improved inter-pregnancy care (45%). Other benefits included using the DIP Clinical Register as a quality assurance tool for DIP services (41%) and using information from the register to assist planning of future services (42%).

### Focus Groups

The primary findings from the focus groups highlighted the complex and fragmented models of care for DIP across regions and organizations in Far North Queensland. The health system is impacted by a range of factors including multiple information systems, disjointed communication, challenges with logistics and access to care in a service which spans a very large demographic.

#### Communication and Information Systems

Participants described challenges in communication between health professionals and organizations, with inconsistent access to electronic medical records and a heavy reliance on emails, facsimile and hand-held records. An Indigenous Health Worker described how:

*There's no information sharing. You can't just hop on to our computer system, you rely onsomeone […] put[ting] you into the correspondence or contact[ing] you*.

Emailing handheld records to the antenatal clinic was one strategy suggested by a midwife for overcoming disjointed communication because “*email gets checked by whoever is on, every day.”* Yet, problems were described with email and facsimile with one medical officer highlighting variability in information transfer being dependent on “*if there's a really diligent midwife.”* A diabetes educator suggested “*communication processes”* could be improved “*just by having generic emails, so it's not person-dependent.”*

Concerns around confidentiality were raised, including whether information was being delivered to the intended recipient. A midwife commented “*I don't know where it's going”* and often “*we don't [receive the information].”* Furthermore, reference was made to the “*legalities around sending secure information by email”* and that “*we actually have a policy […] we are not allowed to email”* [diabetes educator/midwife 1].

Hand-held records are relied upon in some settings. However, Midwife 1 described how “*most women choose not to carry [their hand-held record]”* which creates “*another big problem for us.”* As reflected on by another midwife, this is “*really hard”* as everything has “*to be duplicated and kept the same […] trying to make sure they're all correct in each location.”* Midwife 1 summarized the state of current information systems and said that they are “*hoping to eventually get rid of the written disaster and go with an electronic record.”*

#### Care-Coordination

The inconsistencies and fragmentation of communication and IT systems were reported on as negatively impacting care coordination. Women in remote and many regional areas are often transferred to Cairns to access specialist care and to birth. However, issues with care coordination and lack of systematic processes for referrals and discharge summaries arise from communication breakdown. Midwife 2 described how in their organization “*there's no discharge summary, I don't know whether she had a normal birth, caesarean, and I'm doing a post-natal visit on her and she's been back here for a month.”* Similarly, health professionals in Cairns described a lack of information transfer from referring health professionals in other regions. For example, a diabetes educator said that for “*anyone coming down from the Cape, I don't know when they're coming down.”* Despite this, one Medical Officer reported how “*in the last half a year”* improvements have been made to patient summaries, including “*any changes to medications”* which contrasts to previously when “*the information we g[o]t back wouldn't be very good.”*

The recent introduction of the nurse navigator role at Cairns Hospital for women with GDM aims to improve care coordination, particularly for vulnerable women. As articulated by a midwife, this role will “*help [disengaged women] to access services,”* advocate for their needs and “*coordinate their care […] getting them extra supports”* as required.

#### Impact of Workforce on Care-Coordination

The high turnover of health staff was frequently identified by participants as a workforce challenge impacting on the delivery of care. A diabetes educator/midwife in Cairns said that care coordination “*does fall over every couple of years […] it's change of staffing, all that sort of stuff.”* Again, care-coordination is fraught with issues around person dependence and how when “*health professionals go on leave and [have] not […] been replaced, you might be putting that referral through but it could be months before the client is seen”* [diabetes educator].

Effective care coordination was often described in relation to health professionals' time in their roles. For example, a dietitian described how some “c*ommunities in the Cape have [a] really strong health worker workforce and have had senior health workers in those positions for decades.”* They explained how the implication of this is that “*there's really strong relationships with community”* which, as articulated by an Indigenous Health worker, is important to “*build[ing] that relationship and that trust with the girls [making] follow-ups […] easy*.”

#### Logistics and Access

Given the remote context of FNQ, many women are required to travel to access care which can create challenges. An Obstetrician described that:

“*The main reason why [women] don't come is because they're reluctant to leave their children behind […] or they want to be able to bring them down [but] they need someone with them who can care for their children when they're birthing […] Or some women are […] scared and they don't want to be in Cairns alone.”*

As reported by an Indigenous Health Worker, women “*can have an escort […] in the last four weeks […] of their first pregnancy”* which may overcome some barriers, however, for subsequent pregnancies “*financially it is horrendous”* and “*the cost of flights astronomical.”* Furthermore, as explained by an outreach midwife, the lack of formal care coordination often results in women “*get[ting] a bit lost in the system.”* In town, transport services offered were generally described as being sufficient, with travel officers often facilitating this process, although not all women have access to these services. Another barrier to care was clinic waiting times at the hospital and primary care clinics which can be “*a huge deterrent”* for women. One midwife described how:

“*I've had so many [clients] that have walked out, they've been waiting and waiting.”*

Additional barriers to accessing care include appropriateness of accommodation, access to food, financial security. As summarized by a midwife navigator:

“*If you overcome a lot of those barriers and also got women to come down and relocate, thatdoesn't necessarily guarantee they're still going to engage with services.”*

Support workers were described as being critical to enhancing all women's access to care.

#### Knowledge of Guidelines

Women with DIP are a high priority for health professionals in FNQ. As described by an Outreach Diabetes Educator, “*someone with an abnormal BMI […] or GDM or type 2, […] become a higher priority [than other women].”*

Many health professionals reported adherence to local clinical guidelines in the antenatal period. For example, one midwife commented on how “*most [women] get a HbA1C on their first bloods.”* However, screening practices in the post-partum period are more varied and challenged by disjointed care-coordination, which:

“*…can be tricky because [women] might be coming back for their annual health checks, but if it's not documented in the chart that she had diabetes then it's not done […] we should be able to [enter information in to the electronic medical record] that she needs that annual follow-up forever, but at the moment we don't have the capacity to do that.”*

Midwife

## Discussion

This mixed methods study revealed the complexities and challenges faced by multidisciplinary services working across large geographic locations, which are relevant to many areas of Australia and other countries with similar high-risk populations. It was apparent that caring for women with DIP was a high priority for the health professionals who engaged in the workshops and survey. Issues were similar to those previously identified in the Northern Territory (13) although there were differences in priorities. The key findings were that disjointed communication disrupts the current system, largely because of information technology difficulties. This in turn affects care-coordination along with other factors such as high staff turnover and remoteness. Knowledge of guidelines and screening recommendations as reported by participants was reasonable but selected areas could be improved.

A common theme regarding communication was the variance in electronic and paper records, with multiple services using different platforms that do not interact with each other. This leads to information being lost or communicated to the wrong health provider particularly when women live rurally and seek care from multiple practices. Practitioners therefore often rely on personal communication which is unsustainable when there is high staff turnover. One suggested solution was that of generic email addresses for midwives at a certain location rather than one person as the sole recipient of communications.

Better integration of systems is a common and ongoing challenge, similar to previous reports from the NT, a comparable geographical setting (16). Queensland Health have recognized the need for better integration at a State-wide level and have introduced the Integrated Electronic Medical Record at Cairns Hospital, a “scalable, reliable, and flexible information-sharing capability that allows integration with new and existing systems, across care settings” (21). However, Aboriginal Controlled Community Health Organizations, private practice, and Queensland Health clinics outside of Cairns Hospital are still not able to fully access the hospital electronic record, although there is a staged plan to increase access. Generating a diabetes related discharge summary from the DIP Clinical Register is an innovation which will be trialed by the DIPPINQ to reduce described gaps in communication.

Information technology could also be used more effectively for recall systems. It was evident that many staff involved in pregnancy care were not involved in either pre-pregnancy or postnatal care beyond 6 weeks, or long-term follow-up screening, where an “inter-pregnancy” window to give opportunistic lifestyle and pre-conception counseling exists (22, 23). A need for more structured follow-up systems has been suggested by others (24). The potential for the DIP Clinical Register to generate follow-up lists to inform primary health centers across the region is currently being explored.

Care-coordination is a function of not only electronic communication systems, but also referral pathways and access to care. Our study participants reported that referral pathways were sometimes unclear and that information received from the hospital, in particular, was lacking or untimely. The DIPPINQ models of care component aims to improve transparency of these pathways, by working with local providers to clarify processes in each district. Cairns Hospital has recently created a nurse navigator role, to guide women with GDM through the complexities of multiple appointments, procedures, and travel. This model has been used successfully in other areas and involves a woman-centered intervention using trained personnel to mitigate barriers for women as they access health services, with a particular focus on ameliorating social disadvantage (25, 26). This nurse will work closely with the DIPPINQ team to improve care-coordination. Barriers to healthcare access described by participants included remoteness and cultural factors. It has been reported that using culturally appropriate resources and improving Indigenous workforce involvement are key areas on which to focus (24). One of the main suggestions for improvement was continued education and up-skilling for local health practitioners, including Indigenous Health Workers.

Telehealth is also used routinely in Cairns to assist with the “hub and spoke” model of providing specialist care (18), striving to counter the problems associated with vast distances. Many practitioners were in favor of this approach and thought it could be used more frequently. Telehealth has the potential to improve patient access to health care, reduce travel and inconvenience for patients, families, carers, and health professionals and provide health professionals with access to peer support and education (27). One limitation, however, is that obstetric care including examinations and tertiary level ultrasound scans are not always possible via telehealth. One opportunity identified in both this study and by Edwards et al. (13) was that of increased utilization of telehealth for case conferencing with multiple disciplines to improve care for complex cases.

The majority of health professionals were comfortable with universal screening at 24–28 weeks gestation as per the WHO, ADIPS, and IADPSG (28) guidelines. Who should be screened in early pregnancy still raised uncertainty among the study participants, despite the current routine guidelines being in place for 2 years in the region. This raises the possibility that women at high risk are not being screened appropriately and are subsequently missing out on potential treatment. Participants reported confidence in their knowledge of how to manage DIP, with an exception being education of patients in use of insulin. This may contribute to therapeutic inertia and lack of escalation of treatment from dietary to medical therapy, and has been described in both pregnant (29) and non-pregnant patients with T2DM (30). Future education sessions conducted by DIPPINQ will concentrate on the early screening component of the guidelines and appropriate use of medication in DIP. Empowering midwives in particular to be confident regarding insulin education is a strategy which will be explored. Regular audits and review of data from the DIP Clinical Register by DIPPINQ will assist in assessing whether these strategies are effective.

There were a number of limitations to this study. Study findings may have limited generalizability as invitations to participate were through professional networks and may not have included all relevant health professionals. There was some potential bias in that those who responded were more likely to have an interest in DIP management and the dominant perspective was from midwives, the most represented group. Despite this, roles of other participants were quite varied and located across the geographic regions. Participation was voluntary and the response rate was not obtained. Additionally, some questions may not have been relevant to all health professionals. These issues led to missing data for some of the variables, and this limited our ability to interpret some specific results. Stakeholders had to be available to attend the workshops in person which was difficult for those in rural and remote locations, however, multiple subsequent workshops and telehealth options were offered to be flexible and maximize attendance as much as possible.

The strengths of this study were wide representation of practitioners across urban, rural, and remote settings and participation from many different health professions including Indigenous health workers, doctors, nurses, and allied health professionals. Quantitative data from the survey and qualitative results from the focus groups were comparable and themes were consistent across the two methods. Overall the information gathered was extremely useful to inform priorities for the DIPPINQ models of care work.

## Conclusion

Mapping practitioners' experience providing care for women with diabetes in pregnancy reveals that logistics and management of these women in Far North Queensland can be challenging. There are opportunities for improvement in all the following key themes identified as current concerns: communication systems, information technology, care-coordination, access, and education for health professionals. A complex health systems intervention to address each of these themes is currently being undertaken by the DIPPINQ, with prospective evaluation planned.

## Ethics Statement

Ethics approval for this study was obtained from the Far North Queensland Human Research Ethics Committee (HREC/16/QCH/15).

## Author Contributions

AM: study design, data collection and interpretation, primary author of manuscript. RK: study design, data collection and interpretation, second primary author. SC, CC, JBo, AB, JM, MW, HM, JO, and AS: study design and manuscript review. CW and JBa: data collection and manuscript review. FB: data interpretation and manuscript review. LM-B: study design, data interpretation, manuscript review, and corresponding author.

### Conflict of Interest Statement

The authors declare that the research was conducted in the absence of any commercial or financial relationships that could be construed as a potential conflict of interest.
