# Supplementary material for: Improving Models of Care for Diabetes in Pregnancy: Experience of Current Practice in Far North Queensland, Australia
Source: Front Public Health. 2019 Jul 19;7:192. doi: 10.3389/fpubh.2019.00192 (PMC6659099; doi:10.3389/fpubh.2019.00192)
Supplement: Supplementary file 1 [file Data_Sheet_1.docx]

**Supplement 1:**

**NT & FNQ Diabetes in Pregnancy Partnership Health Professional Survey Questions**

1. **What is your occupation (choose only one to answer this survey)**

Nurse

Midwife

GP

Medical specialist

Other Medical Practitioner

Diabetes Educator

Dietitian

Manager

Indigenous Health Worker

Other (Please specify)

1. **How long have you been in your current position?**

Less than a year

1-5 years

5-10 years

> 10 years

**3. In which region do you live in FNQ?**

Torres Strait Islands and NPA

Cape York

Cairns

Atherton

Mareeba

Innisfail

Yarrabah

Other (please specify)

**4. Do you work in Primary Health Care?**

Yes

No

**5. In which setting do you primarily work?**

Remote

Regional

Urban

Other (please specify)

**6. The women you work with predominantly are:**

Aboriginal women

Torres Strait Islander women

Non-Indigenous women

Both Indigenous and Non-Indigenous women

Other ethnic group

**7. Please rate the following:**

**How confident are you in your own skills to manage women with DIP?**

Not at all confident

Not confident

Neutral

Confident

Extremely confident

**8. Please rate the following :**

**Do you believe most women in your health service or hospital receive appropriate care for diabetes during pregnancy?**

Strongly disagree

Disagree

Neutral

Agree

Strongly agree

**9. Do you routinely screen any women for diabetes early in pregnancy (at first visit or soon after)?**

Yes

No

Unsure

**10. Who do you usually screen for diabetes early in pregnancy at first visit or soon after? ( tick all that apply)**

All pregnant women (no need to select other options)

All Aboriginal and Torres Strait Islander women

Women from other high risk ethnic groups

Women who are older (maternal age > 40 yrs)

Women who are obese

Women with a personal history of GDM or glucose in tolerance

Women with a family history of diabetes or GDM

Women who have had previous pregnancy complications

Women with a history of previous large baby

Women with glycosuria

Women with Polycystic Ovarian Syndrome

Women with hypertension or pregnancy induced hypertension

Women on glucocorticoids (e.g. prednisolone)

Other (please specify)

**11. What screening test do you use most commonly for diabetes early in pregnancy (or at first attendance to a health centre)?**

HbA1c

Fasting BGL

Random BGL

50 gm Glucose Challenge Test

75 gm Glucose Tolerance Test

Unsure

**12. Do you routinely screen for diabetes in the second or third trimester?**

Yes

No

Unsure

**13. Who do you routinely screen for diabetes in the second or third trimester? (tick all that apply)**

□ All Women (no need to select other options)

□ All Aboriginal & Torres Strait Islander women

□ Women from other high risk ethnic groups

□ Women who are older (maternal age >40 yrs)

□ Women who are obese

□ Women with a personal history of GDM or glucose intolerance

□ Women with a family history of diabetes or GDM

□ Women who have had previous pregnancy complications

□ Women with a history of previous large baby

□ Women with glycosuria

□ Women with Polycystic Ovarian Syndrome

□ Women with hypertension or pregnancy induced hypertension

□ Women on glucocorticoids (e.g. prednisolone)

□ Unsure

**14. At what gestation do you aim to screen for diabetes in the second or third trimester?**

24-28 weeks

26-28 weeks

28 weeks

< 24 weeks

> 28 weeks

Other

**15. What screening test do you most commonly use in the second and third trimester?**

HbA1c

Fasting BGL

Random BGL

50 gm Glucose Challenge Test

75 gm Oral Glucose Tolerance Test

Unsure

**16. Do you usually have access to point-of-care (on-site) HbA1c testing equipment (DCA Analyser)?**

Yes

No

Unsure

**17. A. Do you currently use an electronic patient medical record system?**

Yes

No

**B. Do you currently access the My eHealth Record (Shared National Electronic Health Record)?**

Yes

No

**C. Do you promote the My eHealth Record (Shared National Electronic Health Record) to your clients?**

Yes

No

**18. Do you currently use the handheld record (carried by pregnant women)?**

Yes

No

If no, skip to Question 20

**19. Do you currently write in the handheld antenatal record for:**

All women

Indigenous women only

Non- Indigenous women only

Other (Please specify)

**20. Do you ever refer pregnant women to medical specialists (e.g. Endocrinologist, Obstetrician)**

Yes

No

If yes, which specialists

**21. When referring patients to medical specialists do you normally send the referral via (tick all that apply)**

Fax

Directly via electronic record

Phone call to specialist

Email

Mail

Other (Please specify)

**22. In regard to referrals to medical specialists, please rate the following:**

**A. How satisfied are you with the process of referring to medical specialists?**

Very dissatisfied

Dissatisfied

Neutral

Satisfied

Very satisfied

Non-applicable

**B. How satisfied are you with the communication you receive back from medical specialists?**

Very dissatisfied

Dissatisfied

Neutral

Satisfied

Very satisfied

Non-applicable

**23. Do you ever refer pregnant women to allied health specialists? (e.g. dietitian, diabetes educator)**

Yes

No

If yes, which allied health specialists

**24. When referring pregnant women to allied health specialists do you normally send the referral via? (tick all that apply)**

Fax

Directly via electronic record

Phone call to specialist

Email

Mail

Other (Please specify)

**25. In regard to referrals to allied health specialists, please rate the following**

**A. How satisfied are you with the process of referring to allied health specialists?**

Very dissatisfied

Dissatisfied

Neutral

Satisfied

Very satisfied

Non-applicable

**B. How satisfied are you with the communication you receive back from allied health specialists?**

Very dissatisfied

Dissatisfied

Neutral

Satisfied

Very satisfied

Non-applicable

**26. Do you have any comments or suggestions on the referral process?**

**27. Have you been involved in telephone or video case conferences for any client condition in your health service or hospital?**

Yes

No

**28**. **How useful have you found telephone or video case conferencing as part of client care?**

Useless

Not much use

Neutral

Useful

Very useful

Non-applicable

**29. In your opinion, telephone or video case conferencing between professionals for management of diabetes in pregnancy should be used …**

Never

Less often

About the same

More often

Much more often

**30. Have you ever heard of a clinical register?**

Yes

No

**31. Do you think that a clinical register would useful in FNQ with assisting in providing individual care for women with diabetes in pregnancy?**

Yes: Why?

No: Why not?

**32. What role do you see such a clinical register having in your workplace?**

**33. What do you consider to be the main benefits of implementing a clinical register in FNQ for diabetes in pregnancy? (tick all that apply)**

Improved care coordination

Ability to review care provided by other providers

Follow-up screening

Inter-pregnancy care

Pre-conception counselling

Quality Assurance program for DIP services

Planning future DIP services

Other (Please specify)

**34. What do you see as being the main challenges in implementing a register?**

**35. If there was a clinical register for diabetes in pregnancy in FNQ, what information would be useful for you to know about women who are currently pregnant or post-partum? (tick all that apply)**

□ Past obstetric history

□ Current DIP management

□ Latest clinical review

□ Post-partum follow-up

□ Other (Please specify)

**36. How would this information help you in your current role?**

**37. What professional educational resources do you use on a regular basis? (tick all that apply)**

□ Medical databases (e.g. PubMed, Medline)

□ Textbooks

□ Primary Clinical Care Manual and Chronic Disease Manual

□ Queensland Health Clinical Guidelines

□ UpToDate Online

□ Rural Health Education Foundation

□ Other specialist websites
□ Qld Statewide Clinical Networks – (Maternity and Neonatal, Diabetes)

□ Australasian Diabetes in Pregnancy Society guidelines

□ NHMRC antenatal guidelines

□ Other (please specify)

**38. For client education on any topic how often do you use pamphlets, brochures, flip-charts or online resources?**

Never

Occasionally

Sometimes (monthly)

Often (weekly)

Frequently (a few times a week)

**39. For diabetes in pregnancy, which pamphlets, brochures or flip-charts do you use? (tick all that apply)**

□ Queensland Department of Health resources

□ Cairns Diabetes Centre resources

□ Healthy Living NT flipchart or poster

□ Australasian Diabetes in Pregnancy Society resources

□ Diabetes Queensland Resources

□ Indigenous Health Infonet resources

□ Other (Please specify)

**40. What is the most convenient way for you to utilise client educational resources?**

Physically stored in clinic or carried with you

Online (available to download and print as required)

USB stick

DVD

Other (Please specify)

**41. What is your preference for professional education about diabetes in pregnancy?**

□ Course

□ Lecture or talk by specialist

□ Online modules

□ Personal Learning (e.g. journal articles, textbook)

□ As a part of a conference or symposium

**42. Approximately what percentage of the pregnant women you work with have you seen prior to their pregnancy specifically for pre-pregnancy counselling?**

0-20%

20-40%

40-60%

60-80%

80-100%

**43. Approximately what percentage of pregnant women you work with do you see post-partum for ongoing clinical care? (e.g. 6 week check)**

0-20%

20-40%

40-60%

60-80%

80-100%

**44. Do you think medical specialists (e.g. Endocrinologists, Obstetricians) should be involved in managing women with diabetes in pregnancy…**

A lot less

Less

About the same

More

A lot more

**45. Please complete the following for diabetes in pregnancy management**

**A. How confident are you in providing general lifestyle advice (e.g. smoking, exercise, infection
 prevention) to pregnant women with diabetes?**

Not at all confident

Not confident

Neutral

Confident

Very confident

Not applicable

**B. How confident are you in providing education about dietary management for pregnant women with
 diabetes?**

Not at all confident

Not confident

Neutral

Confident

Very confident

Not applicable

**C. How confident are you in providing education about monitoring of blood glucose levels to pregnant
 women?**

Not at all confident

Not confident

Neutral

Confident

Very confident

Not applicable

**D. How confident are you in providing education about administration and storage of insulin?**

Not at all confident

Not confident

Neutral

Confident

Very confident

Not applicable

**46. The following question relates to specialist or hospital appointments and admissions…**

**A. How satisfied are you with the written information you receive from client hospital admissions?**

Very dissatisfied

Dissatisfied

Neutral

Satisfied

Very satisfied

**B. How satisfied are you with the timeliness of information you receive from client hospital
 appointments or admissions?**

Very dissatisfied

Dissatisfied

Neutral

Satisfied

Very satisfied

**C. How satisfied are you with the process of arranging appointments in the nearest hospital or specialist
 clinic?**

Very dissatisfied

Dissatisfied

Neutral

Satisfied

Very satisfied

**47. Which of the following health professionals involved in DIP visit your community on a regular basis (at least every 6 months)? (Tick all that apply)**

□ Endocrinologist

□ Obstetrician

□ Diabetes Educator

□ Dietitian

□ Other (Please specify)

**48. Do you think women with diabetes in pregnancy would benefit from any of the following? (Tick all that apply)**

□ Improved education for local health workers

□ More visiting health professionals

□ More telephone or video case conferences

□ More patient visits to regional centre for appointments

□ Other (Please specify)
